# Feasibility of non-routine abdominal drainage for minimally invasive liver surgery: study protocol for a multicenter randomized controlled clinical trial

**DOI:** 10.1097/SP9.0000000000000049

**Published:** 2025-05-27

**Authors:** Shohei Yoshiya, Shinji Itoh, Mizuki Ninomiya, Keishi Sugimachi, Kazutoyo Morita, Noboru Harada, Hideaki Uchiyama, Kengo Fukuzawa, Toru Utsunomiya, Takashi Maeda, Ryosuke Minagawa, Mototsugu Shimokawa, Tomoharu Yoshizumi

**Affiliations:** a Department of Surgery and Science, Graduate School of Medical Sciences, Kyushu University, Fukuoka, Japan; b Department of Surgery, Oita Red Cross Hospital, Oita, Japan; c Department of Surgery, Iizuka Hospital, Fukuoka, Japan; d Department of Hepato-Biliary-Pancreatic Surgery, National Hospital Organization Kyushu Cancer Center, Fukuoka, Japan; e Department of Liver Surgery, Fukuoka City Hospital, Fukuoka, Japan; f Department of Surgery, Saiseikai Fukuoka General Hospital, Fukuoka, Japan; g Department of Hepato-Biliary-Pancreatic Surgery, National Hospital Organization Fukuoka Higashi Medical Center, Fukuoka, Japan; h Department of Surgery, Oita Prefectural Hospital, Oita, Japan; i Department of Hepato-Biliary-Pancreatic Surgery, Hiroshima Red Cross Hospital & Atomic-Bomb Survivors Hospital, Hiroshima, Japan; j Department of Hepato-Biliary-Pancreatic Surgery, Matsuyama Red Cross Hospital, Ehime, Japan; k Department of Biostatistics, Yamaguchi University Graduate School of Medicine, Yamaguchi, Japan

**Keywords:** drainage, hepatectomy, hepatic resection, liver resection, minimally invasive surgery

## Abstract

**Introduction:**

Minimally invasive liver surgery (MILS), such as laparoscopic and robotic hepatectomy, has been developed and is an effective alternative to traditional open hepatectomy. Although surgical techniques and postoperative management have improved, many institutions continue to perform routine postoperative abdominal drainage. In open hepatectomy, abdominal drainage after uncomplicated hepatectomy increases overall and wound-related complications without a reduction in the risk of intra-abdominal fluid collections that require intervention. The aim of this study was to elucidate the feasibility of non-routine abdominal drainage for patients who undergo MILS for tumors located outside the posterosuperior area.

**Methods and analysis:**

This study is a multicenter randomized controlled trial and will recruit 182 patients who undergo MILS. The study duration is three years, including a 2-year registration duration. Participants will be randomly assigned to either the non-routine drainage group or the routine drainage group (ratio 1:1) to prove non-inferiority. The primary study outcome is the incidence of in-hospital postoperative complications of Clavien–Dindo grade ≥ II. The secondary study outcomes are length of postoperative hospital stay, incidences of specific postoperative complications and all postoperative complications, and surgery-related mortality.

**Ethics and dissemination:**

Ethical approval has been obtained from the institutional review board (No. 20232008). The results of this study will be published in international peer-reviewed journals.

## Introduction

Although the outcomes of other treatment modalities, mainly chemotherapy with or without immune checkpoint inhibitors, have recently improved, surgical hepatic resection for liver tumors, including hepatocellular carcinoma, colorectal liver metastases, and cholangiocarcinoma, remains the cornerstone of the treatment strategy^[1-4]^. Curative liver resection for a primary or metastatic liver tumor could lead to a favorable long-term prognosis after surgery^[5,6]^. Moreover, early recovery from surgical invasiveness is considered important and is the basis for the common use of enhanced recovery after surgery protocols[7].

To reduce physical damage and to accelerate postoperative recovery, minimally invasive liver surgery (MILS), such as laparoscopic and robotic hepatectomy, has been developed and is an effective alternative to traditional open hepatectomy. Several studies have reported that MILS for minor or major liver resection resulted in both short- and long-term oncological favorable outcomes^[4,8,9]^.

Although surgical techniques and postoperative management have improved, it is presumed that many institutions continue to perform routine abdominal drainage to facilitate early diagnosis of postoperative bleeding or bile leakage, and to potentially prevent intra-abdominal fluid collections^[10,11]^. Notably, early drain removal after hepatectomy has been included in enhanced recovery after surgery guidelines and may be associated with faster recovery and reduced length of hospital stay[12]. In open hepatectomy, seven randomized controlled trials (RCTs)^[13-19]^ and a meta-analysis of these RCTs[20] have been published. The meta-analysis found that abdominal drainage after uncomplicated hepatectomy increased overall and wound-related complications without a reduction in the risk of intra-abdominal fluid collections that required intervention[20]. On the other hand, there has been no RCT in MILS.

## Objective

The objective of this multicenter randomized comparative study is to elucidate the feasibility of non-routine abdominal drainage as non-inferiority for patients who undergo MILS for primary or metastatic liver tumors located outside the posterosuperior area.

## Methods and analysis

### Study design

This study is an RCT (1:1 ratio) in which eligible patients who meet the inclusion criteria will be enrolled and randomly assigned to either the non-routine abdominal drainage group or the routine abdominal drainage group after providing consent. The randomization will be performed using UMIN Internet Data and Information system for Clinical and Epidemiological research, Cloud version with minimization method and assignment factors are institution, diagnosis (primary liver tumor/metastatic liver tumor), and surgical procedure (partial hepatectomy/subsegmentectomy/left lateral segmentectomy). MILS will be performed by laparoscopy or robotic hepatectomy. This is a non-inferiority study to confirm the efficacy and safety of non-routine abdominal drainage compared with routine abdominal drainage in patients who undergo MILS, including S2, S3, S4, S5, and S6 partial hepatectomy; S3, S5, and S6 subsegmentectomy; and left lateral segmentectomy. This study conforms to the CONSORT criteria. The study duration is three years, including a 2-year registration duration.

## Endpoints

Primary study outcome:

Incidence of in-hospital postoperative complications of Clavien-Dindo grade ≥ II resulting from MILS including resulting from drainage.

Secondary study outcomes:

1. Length of postoperative hospital stay;
2. Incidence of specific postoperative complications (i.e. biliary leakage, postoperative bleeding, massive ascites, massive pleural effusion, surgical site infection);
3. Incidence of all postoperative complications;
4. Surgery-related mortality.

## Patient eligibility

Inclusion criteria: Patients who meet all of the following criteria will be eligible:

1. Primary or metastatic liver tumors that are eligible for laparoscopic hepatectomy or robot-assisted laparoscopic hepatectomy at the participating institution from the date of study approval;
2. S2, S3, S4, S5, or S6 partial hepatectomy; S3, S5, or S6 subsegmentectomy; or left lateral segmentectomy as the surgical procedure;
3. Eastern Cooperative Oncology Group performance status grade 0–1;
4. Patients who have received a full explanation of the study and provide their free and voluntary consent based on their full understanding of the study.

Exclusion criteria: Patients will be excluded if they meet any of the following criteria:

1. Complicated resection of other organs, excluding the gallbladder;
2. Serious comorbidities;
3. Did not agree to participate in the study after receiving a full explanation of the study;
4. Deemed inappropriate for inclusion by the investigator.

## Surgical procedures

All participating centers are high-volume centers for hepatobiliary and pancreatic surgery. The treating surgeons at all participating centers are beyond their learning curves for performing MILS. The surgical procedure, selection of robotic or laparoscopic hepatectomy, extent of hepatic resection, and the method of hepatic parenchymal transection (clamp-crushing method or Cavitron ultrasonic surgical aspirator), will be determined by each investigator in accordance with their institutional criteria. In the routine drainage group, after completion of liver resection, a single silicone drain will be placed under the cut surface of the liver, or in the subphrenic space or Winslow’s foramen at the end of surgery; the other end will be pulled through a skin incision and connected to a closed bag with suction. The drain will be removed in accordance with each institution’s criteria depending on the amount of drainage, without bile leakage findings (the International Study Group of Liver Surgery definition[21]). On the contrary, in the non-routine group, after completion of liver resection, the abdomen is closed without insertion of a silicone drain, and the operation is terminated.

## Data collection

This study will examine and collect the information listed below and in Table 1. Blood tests and abdominal contrast-enhanced computed tomography (CT) will be routine procedures performed to collect standard information, as in regular medical care.

- Patient background information: sex, age, body mass index, primary disease, liver function prior to surgery, surgical history;
- Surgical factors: surgical procedure, duration of surgery, blood loss, duration of the Pringle maneuver, pathological liver fibrosis;
- Blood tests: hematological indices, liver function indices, kidney function indices;
- Ascites biochemical testing: bilirubin level in the drainage fluid, including new punctures;
- Complications: presence of postoperative complications and which ones;
- Abdominal contrast-enhanced CT to evaluate fluid collections or other radiological findings.

**Table 1:** Information that will be collected in this study

Factors	Prior to surgery	Following surgery	Observation period following surgery		Upon discontinuation
Period	0-4 weeks before	0 weeks	1 week following surgery	At discharge	Upon discontinuation
Medical checkup	○	–	○	○	○
Obtainment of consent	○	–	–	–	–
Random allocation	●	–	–	–	–
Patient background	○	–	–	–	–
Surgical factors		○			
Blood test	○		○	○	○
Ascites biochemical test	–	–	○	○	○
Regarding complications	–	–	○	○	○
Abdominal contrast-enhanced CT	○	–	○	–	○

CT, computed tomography.

## Sample size

The primary endpoint of the study is the incidence of postoperative complications of Clavien–Dindo grade ≥ II resulting from hepatic resection, in the full analysis set (FAS). A meta-analysis of open hepatectomy with and without routine abdominal drainage showed that the rate of postoperative complications of Clavien–Dindo grade ≥ II was 36.1% in the routine drainage group, 26.6% in the non-routine drainage group, and 32.6% in all patients[22]. Additionally, the odds ratio for postoperative complications associated with MILS versus open hepatectomy was approximately 0.5[23]. The anticipated complications rates for MILS in this study are 20% in the routine drainage group and 15% in the non-routine drainage group. Although the overall complications rate in a study of non-routine drainage in open hepatectomy was 32.6%[22], it is clear that the complications rate for MILS is lower than that for open hepatectomy, and the complications rate with non-routine drainage in MILS should be lower than that for open hepatectomy. Therefore, it is considered clinically acceptable if the complications rate in the non-routine drainage group is no more than 10% higher than that in the routine drainage group. Therefore, we set the non-inferiority margin at 10%, and calculated the required number of patients for each group as 82, assuming a one-sided alpha error of 5% and a power of 80%. Considering an expected dropout rate of approximately 10%, we set the target number of patients at 182 in total (91 cases per group).

## Analysis set

FAS: Patients who have received all or part of the protocol treatment, excluding ineligible patients, and patients who have undergone at least one efficacy evaluation. However, patients who are found ineligible after enrollment will be excluded.

Per protocol set: Patients included in the FAS who are not in serious non-compliance with the study protocol.

Safety set: The group of patients for whom some or all of the protocol treatment has been administered.

## Statistical analysis

The non-inferiority of the primary study endpoint between the non-routine abdominal drainage and routine abdominal drainage groups will be met if the upper limit of the 90% confidence interval for the risk difference, calculated using the Cochran–Mantel–Haenszel method with allocation-adjusting factors as covariates, is within the non-inferiority margin of 10%. No interim analyses are planned.

## Discussion

Surgical hepatectomy has long referred to open hepatectomy, but since the introduction of laparoscopic hepatectomy, the percentage of laparoscopic hepatectomies has increased over time in many institutions. Many studies have confirmed the safety and equality of short- and long-term postoperative outcomes of laparoscopic compared with open hepatectomy^[4,23]^. Regarding postoperative complications, there are lower odds of experiencing all complications (odds ratio: 0.42) and severe complications (odds ratio: 0.51) for patients who undergo laparoscopic versus open hepatectomy[23]. Additionally, although the safety and efficacy of MILS for technically complicated procedures has been confirmed^[24,25]^, these results remain controversial in difficult cases. Robotic hepatectomy was recently introduced, and there are scattered reports on the usefulness of this approach. Recent meta-analysis also revealed that the robotic approach had advantages to laparoscopic in terms of lower blood loss and reduced rates of open conversion, especially in difficult hepatectomies[26]. Therefore, MILS is likely to become even more widely used internationally in the treatment of liver tumors.

Several RCTs and a meta-analysis of these RCTs found that abdominal drainage after hepatectomy increased the incidence of overall and wound-related complications^[20,22]^. These studies suggested that surgeons avoid routine drainage after uncomplicated hepatectomy; however, routine abdominal drainage continues to be performed after hepatectomy in many institutions. The drain is usually placed on the cut surface of the liver, or in the subphrenic space or Winslow’s foramen to detect and treat bile leakage, monitor postoperative intra-abdominal bleeding, or relieve intra-abdominal tension due to ascitic fluid collections^[11,20]^. Most RCTs evaluated drainage in open hepatectomy, which may explain why surgeons have not focused on the results of these studies. Therefore, we decided to focus exclusively on MILS for tumors located outside the posterosuperior area, which will comprise the majority of liver resections in the future. If our results reveal the feasibility of non-routine abdominal drainage after MILS, this finding could assist surgeons when deciding to place a postoperative abdominal drain.

This study has some limitations that should be considered when interpreting its findings. The generalizability and reproducibility will be concerned because of multicenter study. Multicenter study may have the problems of surgical variability and postoperative management strategy in institutions and data standardization. Also, since this study is planned to have a relatively small number of participants (91 in each group), detailed subgroup analysis may be difficult from a power point of view. Recently, artificial intelligence technology (AI) has provided variable opportunities for medical research[27] and medical diagnostic treatment[28]. AI could improve the generalizability and reproducibility of multicenter study with standardizing the analysis of postoperative data, reducing human error and providing a unified data processing framework. Therefore, further study using AI will provide optimizing drainage strategies in MILS.

## Data Availability

Datasets generated during and/or analyzed during the current study are publicly available upon reasonable request.

## Research registration unique identifying number (UIN)

The study is registered in UMIN-CTR as UMIN000053024.

## References

[1] Reig M Forner A Rimola J. BCLC strategy for prognosis prediction and treatment recommendation: the 2022 update. J Hepatol 2022;76:681–93. 34801630 10.1016/j.jhep.2021.11.018 PMC8866082

[2] Benson AB D’Angelica MI Abbott DE. Hepatobiliary cancers, version 2.2021, NCCN clinical practice guidelines in oncology. J Natl Compr Canc Netw 2021;19:541–65. 34030131 10.6004/jnccn.2021.0022

[3] Yoshiya S Harada N Toshima T. Treatment strategy for hepatocellular carcinoma recurrence in the transplant era: focusing on the Japan criteria. Surg Today 2024;54:64–72. 37289265 10.1007/s00595-023-02710-z

[4] Fichtinger RS Aldrighetti LA Abu Hilal M. Laparoscopic versus open hemihepatectomy: the ORANGE II PLUS multicenter randomized controlled trial. J Clin Oncol Off J Am Soc Clin Oncol 2024;42:1799–809. 10.1200/JCO.23.01019 PMC11107897 38640453

[5] Iseda N Itoh S Toshima T. Outcome of hepatectomy after systemic therapy for hepatocellular carcinoma: a Japanese multicenter study. Surg Today 2024;54:510–17. 10.1007/s00595-024-02930-x 39192138

[6] Yohanathan L Chopra A Simo K. Assessment and treatment considerations for patients with colorectal liver metastases: AHPBA consensus guideline and update for surgeons. HPB (Oxford) 2025; 27:263–78. 39828468 10.1016/j.hpb.2024.12.006

[7] Noba L Rodgers S Chandler C. Enhanced Recovery After Surgery (ERAS) reduces hospital costs and improve clinical outcomes in liver surgery: a systematic review and meta-analysis. J Gastrointest Surg 2020;24:918–32. 31900738 10.1007/s11605-019-04499-0 PMC7165160

[8] Zhang KJ Liang L Diao YK. Short- and long-term outcomes of laparoscopic versus open liver resection for large hepatocellular carcinoma: a propensity score study. Surg Today 2023;53:322–31. 35986784 10.1007/s00595-022-02576-7

[9] Koh YX Zhao Y Tan IE. Cost-effectiveness of laparoscopic vs open liver resection: a propensity score-matched single-center analysis of 920 cases. J Am Coll Surg 2024. 10.1097/xcs.0000000000001250. 39620602

[10] Bona S Gavelli A Huguet C. The role of abdominal drainage after major hepatic resection. Am J Surg 1994;167:593–95. 8209934 10.1016/0002-9610(94)90104-x

[11] Bekki Y Yamashita Y Itoh S. Predictors of the effectiveness of prophylactic drains after hepatic resection. World J Surg 2015;39:2543–49. 26059409 10.1007/s00268-015-3116-3

[12] Burchard PR Dave YA Loria AP. Early postoperative ERAS compliance predicts decreased length of stay and complications following liver resection. HPB 2022;24:1425–32. 35135723 10.1016/j.hpb.2022.01.008

[13] Belghiti J Kabbej M Sauvanet A. Drainage after elective hepatic resection. A randomized trial. Ann Surg 1993;218:748–53. 8257225 10.1097/00000658-199312000-00008 PMC1243070

[14] Fong Y Brennan MF Brown K. Drainage is unnecessary after elective liver resection. Am J Surg 1996;171:158–62. 8554132 10.1016/s0002-9610(99)80092-0

[15] Liu CL Fan ST Lo CM. Abdominal drainage after hepatic resection is contraindicated in patients with chronic liver diseases. Ann Surg 2004;239:194–201. 14745327 10.1097/01.sla.0000109153.71725.8c PMC1356212

[16] Fuster J Llovet JM Garcia-Valdecasas JC. Abdominal drainage after liver resection for hepatocellular carcinoma in cirrhotic patients: a randomized controlled study. Hepatogastroenterology 2004;51:536–40. 15086197

[17] Sun HC Qin LX Lu L. Randomized clinical trial of the effects of abdominal drainage after elective hepatectomy using the crushing clamp method. Br J Surg 2006;93:422–26. 16491462 10.1002/bjs.5260

[18] Kim YI Fujita S Hwang VJ Nagase Y. Comparison of abdominal drainage and no-drainage after elective hepatectomy: a randomized study. Hepatogastroenterology 2014;61:707–11. 26176061

[19] Arita J Sakamaki K Saiura A. Drain placement after uncomplicated hepatic resection increases severe postoperative complication rate: a Japanese multi-institutional randomized controlled trial (ND-trial). Ann Surg 2021;273:224–31. 33064385 10.1097/SLA.0000000000004051

[20] Hajibandeh S Hajibandeh S Raza SS. Abdominal drainage is contraindicated after uncomplicated hepatectomy: results of a meta-analysis of randomized controlled trials. Surgery 2023;173:401–11. 36424196 10.1016/j.surg.2022.10.023

[21] Rahbari NN Elbers H Koch M. Bilirubin level in the drainage fluid is an early and independent predictor of clinically relevant bile leakage after hepatic resection. Surgery 2012;152:821–31. 22657729 10.1016/j.surg.2012.03.012

[22] Anweier N Apaer S Zeng Q. Is routine abdominal drainage necessary for patients undergoing elective hepatectomy? A protocol for systematic review and meta-analysis. Medicine (Baltimore) 2021;100:e24689. 33578602 10.1097/MD.0000000000024689 PMC10545016

[23] Haney CM Studier-Fischer A Probst P. A systematic review and meta-analysis of randomized controlled trials comparing laparoscopic and open liver resection. HPB 2021;23:1467–81. 33820689 10.1016/j.hpb.2021.03.006

[24] Loke S Ong BDC Ng J Kow AWC. Safety and efficacy of minimally invasive associating liver partition and portal vein ligation for staged hepatectomy (ALPPS): a systematic review and meta-analysis. Int J Surg 2025;111:2283–90. 39869398 10.1097/JS9.0000000000002240

[25] Famularo S Milana F Ardito F. Laparoscopic versus open resection for hepatocellular carcinoma according to the procedure’s complexity: real-world weighted data from a national register. HPB 2024. doi:10.1016/j.hpb.2024.12.017. 39800599

[26] Wang P Zhang D Huang B. Robotic versus laparoscopic hepatectomy: meta-analysis of propensity-score matched studies. BJS Open 2025;9:zrae141. 40164991 10.1093/bjsopen/zrae141 PMC11957917

[27] Guo SB Meng Y Lin L. Artificial intelligence alphafold model for molecular biology and drug discovery: a machine-learning-driven informatics investigation. Mol Cancer 2024;23:223. 39369244 10.1186/s12943-024-02140-6 PMC11452995

[28] Guo SB Cai XY Meng Y. AI model using clinical images for genomic prediction and tailored treatment in patients with cancer. Lancet Oncol 2025;26:e126. 40049202 10.1016/S1470-2045(25)00008-7

